# Prevalence and characteristics of mouse mammary tumor virus-like virus associated breast cancer in China

**DOI:** 10.1186/s13027-021-00383-2

**Published:** 2021-06-26

**Authors:** Fa-liang Wang, Xiao-li Zhang, Ming Yang, Jun Lin, Yong-fang Yue, Ya-dan Li, Xian Wang, Qiang Shu, Hong-chuan Jin

**Affiliations:** 1 Department of Surgical Oncology, The Children’s Hospital, Zhejiang University School of Medicine, National Clinical Research Center for Child Health, Binsheng Road 3333, Hangzhou, 310052 China; 2 Electron Microscope Room, Medical School of Hebei North University, Zhangjiakou, China; 3 Department of Medical Oncology, the Second People’s Hospital of Jande, Hangzhou, China; 4 Department of Obstetrics and Gynecology, the Women’s Hospital, School of Medicine, Zhejiang University, Hangzhou, China; 5 Laboratory of Cancer Biology, Department of Medical Oncology, Key lab of Biotherapy in Zhejiang, Sir Run Run Shaw Hospital, School of Medicine, Zhejiang University, East Qingchun Road 3, Hangzhou, 310000 China; 6 Department of Cardiothoracic Surgery, The Children’s Hospital, Zhejiang University School of Medicine, National Clinical Research Center for Child Health, Binsheng Road 3333, Hangzhou, 310052 China

**Keywords:** Mouse mammary tumor virus-like virus (MMTV-LV), Breast cancer, M. domesticus mouse, M.musculus mouse, M.castaneus mouse

## Abstract

**Background:**

Despite extensive molecular epidemiological studies, the prevalence and characteristics of Mouse Mammary Tumor Virus-Like Virus (MMTV-LV) in Chinese women breast cancer are still unclear. Besides, the prevalence of MMTV-LV in women breast cancer tissue varies in different countries and its dependent factors remain inconclusive.

**Methods:**

In the first part of the study, a case-control study was performed. 119 breast cancer samples (84 from Northern China and 35 from Southern China) and 50 breast fibroadenoma specimens were collected from Chinese women patients. MMTV-like env sequence and the homology to MMTV env gene were analysed by semi-nested polymerase chain reaction (PCR). We also explored the association of MMTV-LV prevalence with sample sources (Southern and Northern China) and patients’ clinicopathological characteristics. To investigate the dependent factors of the prevalence of MMTV-LV in breast cancer worldwide, a meta-analysis was conducted in the second part of the study.

**Results:**

We found that the prevalence of MMTV-LV was much higher in breast cancer tissues (17.65%) than that in breast fibroadenoma specimens (4.00%) (*P* < 0.05). MMTV-LV prevalence in Chinese women breast cancer tissues was significantly different between Southern China (5.71%) and Northern China (22.62%) (*P* < 0.05). The prevalence of MMTV-LV also associates significantly with expression of HER2, but shows no significant correlation with other parameters. In the meta-analysis, we found that MMTV-LV prevalence in breast cancer tissue was dependent on the distribution of M. domesticus mouse (M. d), M. musculus mouse (M.m) and M.castaneus mouse (M.c) worldwide (*P* < 0.05).

**Conclusion:**

The distribution of house mice may be a crucial environmental factor that explains the geographic differences in human breast cancer incidence. Our findings may provide a potential avenue of prevention, diagnosis and treatment for breast cancer.

**Supplementary Information:**

The online version contains supplementary material available at 10.1186/s13027-021-00383-2.

## Introduction

Breast carcinoma is a leading cause of cancer mortality in women worldwide. Many risk factors have been identified in the development of this tumor, such as diet, obesity, nulliparity, hormones, increased life expectancy and family history [1–6]. However, the etiology and molecular mechanism of breast carcinogenesis remain inconclusive despite decades of research [7, 8].

Increasing evidence suggests that viral infection may play a key role in the pathogenesis of breast cancer [9]. As early as 1936, John Bittner discovered the “mouse milk factor” that could increase the incidence of mouse breast cancer [10]. The “factor” was proved to be the mouse mammary tumor virus (MMTV) in the subsequent studies [11–13]. Subsequently, MMTV-like antigens and viral particles were successively detected in human milk and cancer cells during the 1970s and 1980s [14, 15]. In the recent several years, studies using PCR method with primers from selected regions of the MMTV env gene screened MMTV-like sequences in human milk [13] and breast cancer specimens [16–23]. Up to now, various investigators have postulated that mouse mammary tumor virus-like virus (MMTV-LV), a similar virus to MMTV, might be a cause for human breast cancer [24–27].

MMTV-LV prevalence in human breast cancer varies with geographical location. In recent studies, MMTV-LV prevalence was 30–40% in breast cancer tissues from several Western and North Africa countries including America, Italy, Mexico, Australia, Argentina and Tunisia [16, 17, 19, 24, 25, 28–45]. In East and Southeast Asia, where breast cancer incidence is known to be low, the presence of MMTV-LV sequences in breast cancer tissues is 16.79% in China [46]; while the ratio is 0.62 and 1.72% in Vietnam and Myanmar respectively [34, 47].

House mice, the carriers of MMTV, also distribute differently all over the world. Sage et al. divided them into three subspecies, namely M. domesticus (M.d), M.musculus (M.m) and M.castaneus (M.c) [48]. M. d mice originally landed from North Africa to Western Europe, and subsequently migrated to America, Australia, New Zealand and Hawaii via ships sailing from western European ports. While M. m mice distribute along with the range of M. d from Central Europe to East Asia and M.c settles from Southern China to central Iran [48, 49]. The ability to transmit MMTV varies in the three species of house mice due to the different copy number of MMTV in these mice [49].

It’s still unclear whether MMTV-LV has been transferred to humans from mice. If so, the prevalence of MMTV-LV in human breast cancer should be correlated with the distribution of house mice. Accordingly, the prevalence of MMTV-LV in breast cancer should be different between Southern and Northern China, and vary in countries with different house mice distribution.

In this study, we firstly evaluated the different prevalence of MMTV-LV in human breast cancer in Southern and Northern China using a case-control study. And then we analyzed the possible association between the presence of MMTV-LV sequences and various Clinicopathological parameters. Finally, we pooled all the data related to MMTV-LV prevalence in breast cancer worldwide together and conducted meta-analysis and meta-regression to assess the effect of house mouse distribution on MMTV-LV prevalence.

## Materials and methods

### Case-control study

#### Patients and specimens

Paraffin embedded formalin fixed tissues from 169 Chinese women (119 breast cancer patients and 50 breast fibroadenoma patients) were obtained from samples collected between 2005 and 2019 in the Department of Pathology at the Second People’s Hospital of Jiande and the First Affiliated Hospital of Hebei North University, which locate in Southern and Northern China, respectively. Since the project was a retrospective study and used the remaining clinical samples, informed consent was exempted. The ethical issues were approved by Medical Ethics Committee of the Second People’s Hospital of Jiande (Supplementary Fig. 1). 35 breast cancer samples were collected from Chinese women who lived in Southern China, and the other 84 breast cancer samples were from Northern China. WHO criterias were adopted in histopathological diagnosis of each tumor [50]. The distribution of tumors was as follows: 107 invasive ductal carcinoma, 9 invasive lobular carcinoma and 3 mucous adenocarcinoma. According to the modified Scarff-Bloom-Richardson (SBR) system, invasive ductal carcinomas were graded into three grades [51]. The clinicopathological characters of the patients were summarized in Table 1.

**Table 1:** Clinicopathological characteristics of breast cancer patients

Variable	Number of cases (%)
Total	119
Age (years)	
<35	3 (2.52)
35–50	59 (49.58)
>50	57 (47.90)
Sample source (province)	
Hebei	84 (70.59)
Zhejiang	35 (29.41)
Histological type	
Invasive ductal carcinoma	107 (89.92)
Invasive lobular carcinoma	9 (7.56)
Mucious Adenocarcinoma	3 (2.52)
Histological grade ^a^	
Grade I	48 (40.34)
Grade II	49 (41.18)
Grade III	10 (8.40)
Lymph node involvement	
Negative	60 (50.42)
Positive	59 (49.58)

^a^ Histological grade was performed only in specimens with invasive ductal carcinoma

#### DNA extractions and PCR

Formalin fixed paraffin embedded tissue were scraped from paraffin blocks using Ultra-Thin Semiautomatic Microtome and transferred to 1.5 ml eppendorf tubes using bamboo sticks. To avoid cross contamination, bamboo sticks were used for each paraffin block. TIANamp Genomic DNA Kit was used to extract genomic DNA. The sections scraped were deparaffinized in 1 ml xylene and centrifuged at 12,000 g for 3 min. After repeating this step twice, the sections were immersed twice in 1 ml absolute ethanol to remove xylene. Other steps were according to the manufacturer’s instructions. DNA from breast cell lines of MCF7, Bcap37, MDA-MB-231and MDA-MB-453 were also extracted using the TIANamp Genomic DNA Kit under the manufacturer’s guidance.

The semi-nested PCR method with excellent sensitivity, specificity and simplicity was adopted to detection of MMTV env gene-like sequences [37]. 200 ng of genomic DNA was used as template in the first-stage of PCR with outer primers J948-env-F (5′-CCTCACTGCCAGATCGCCTT-3′) and J950-env-R (5′-CAGGTAGCAGCACATATGGC-3′) to amplify a 601 bp DNA fragment. 2ul of the first-stage product was used in the second round PCR as template with the primer pair J948-env-F and J1011-env-R1 (5′-CCTGCTTCATACCATCGATGAACC-3′), amplifying a 254 bp inner sequence (Table 2 and Fig. 2A).

**Table 2:** Primer sequences and location in the MMTV env gene

Designation	Sequence(5′-3′)	Location
J948-env-F	CCTCACTGCCAGATCGCCTT	6048–6067
J950-env-R	CAGGTAGCAGCACATATGGC	6629–6648
J1011-env-R1	CCTGCTTCATACCATCGATGAACC	6278–6301
IDD428-GAPDH-F	GGAGTCAACGGATTTGGT	
IDD429-GAPDH-R	GTGATGGGATTTCCATTGAT	

All PCR amplifications were carried out in a 30 μl reaction system containing 200 mM of each dNTP, 0.2 μM of each forward and reverse primers, 1 U DNA polymerase, 50 mM KCl, 10 mM Tris-HCl (pH 8.4) and 1.5 mM MgCl_2_. Thermocycling was carried out in thermal circulator by denaturing at 94 °C for 5 min, then 35 circles (denaturing at 94 °C for 35 s, annealing at 52 °C for 30 s, extending at 72 °C for 40 s), and finally extending at 72 °C for 10 min. The reproducibility of the PCR reactions was confirmed in triplicate.

**Fig. 1:**
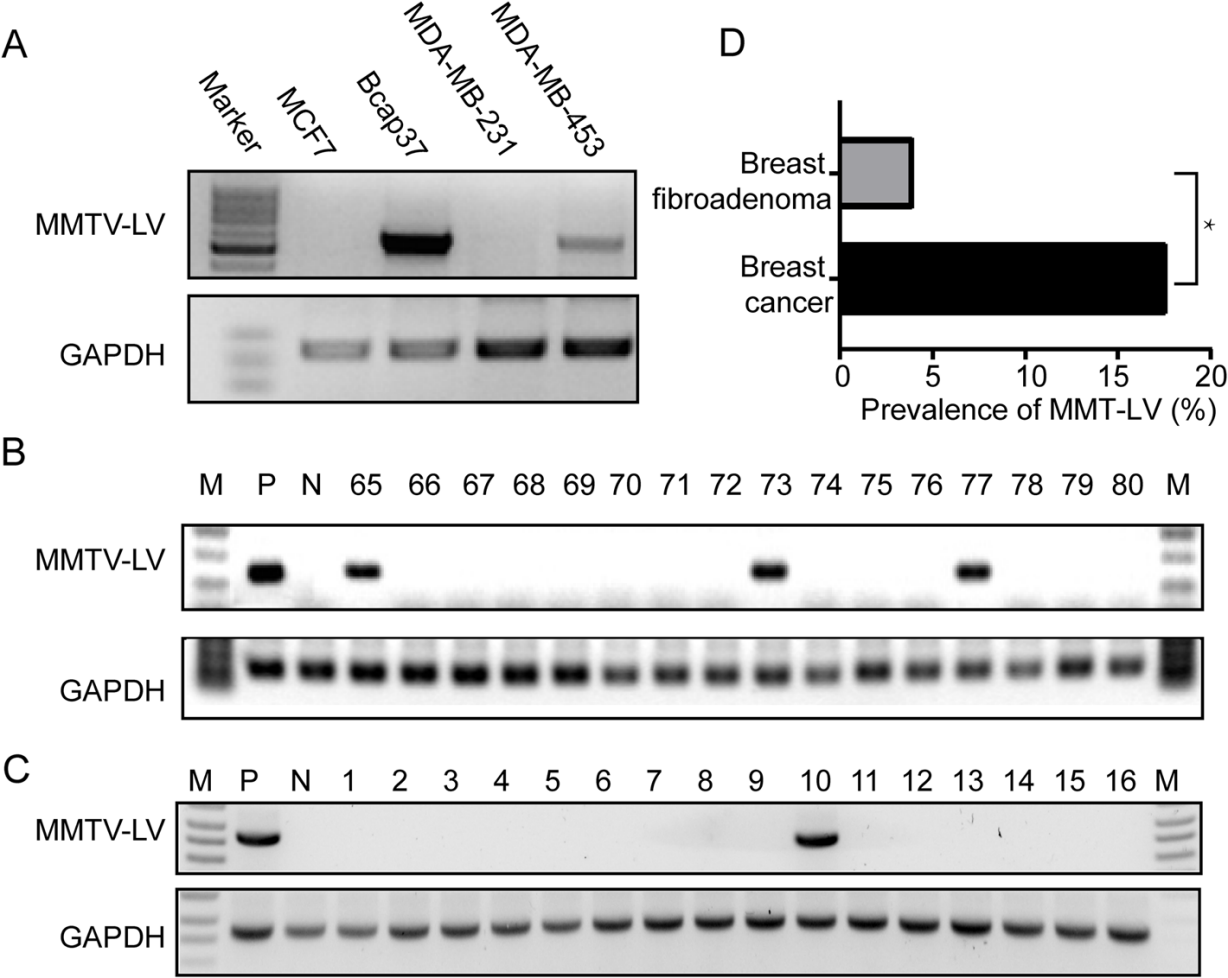
Detection of MMTV-LV sequence in breast cancer. 1.5% agarose gel electrophoresis of the GelRed-stained amplification products for MMTV-LV gene screening using a semi-nested PCR assays. **A**. breast cancer cell lines **B**. breast cancer specimens **C**. breast fibroadenoma tissues **D**. Prevalence of MMTV-like env sequence in Chinese women breast cancer specimens compared to Chinese women breast fibroadenoma tissues (based on Table 3) *Indicating the significant difference between groups for *P* < 0.05

Before detecting the DNA samples from patients, ordinary PCR with primers J948-env-F and J950-env-R was carried out on the DNA from breast carcinoma cell lines MCF7, Bcap37, MDA-MB-231 and MDA-MB-453. MMTV-like env gene sequences were positive in Bcap37 and MDA-MB-453 (Fig. 1C), so we treated DNA from Bcap37 cells as positive control. Negative controls were reactions without adding DNA template. In order to confirm the existence of viral DNA and exclude the amplification of non-specific or endogenous retroviral sequences, twelve randomly selected positive PCR products were purified and sequenced.

#### Immunohistochemical staining and analysis

The expression of Estrogen receptor (ER), Progesterone receptor (PR), and HER2 was detected using immunohistochemistry method. The immunohistochemical staining was interpreted by the same experienced pathologist according to the American Society of Clinical Oncology and College of American Pathologists guidelines, who did not know any other clinicopathological or molecular information. For ER and PR, if 10% or more tumor cells show nuclear staining, the case is considered positive. The HER2 expression was estimated and scored on a scale of 0 to 3+ according to the membrane staining pattern [52].

#### Data analysis

MMTV-LV prevalence in tumors was analyzed for possible association with clinicopathological parameters (age, sample source, histological grade and type, lymph node status and tumor positions) and the expression of receptors (ER, PR and HER2) using χ^2^ or Fisher’s test with SPSS 20.0. If *P* value was less than 0.05, the comparison between groups was considered statistically significant. DNA similarity alignments were performed using DNAMAN 8.0 software.

### Systematic review and meta-analysis

#### Search strategy

A comprehensive search of PubMed, Google Scholar, Web of Science and EBSCO (ASP/BSP) was done to find publications reported between January 1995 and May 2020 using the MeSH terms and key words “mouse mammary tumor virus”, “MMTV” “Human” and “breast cancer or breast carcinoma”. We also evaluated the relevant references cited in retrieved articles.

#### Eligibility criteria

Studies were included according to the following criteria: (i) Studies had to use PCR method to screen MMTV-LV sequence in specimens. Researches were excluded if their PCR product not homologous to MMTV [53]. (ii) Researches only focusing on breast cancer cell line [42] and mice breast tumors [54] were not included. (iii) If data was published in more than one study, only the publication with the most explicit description was included. The article selection process applied in this meta-analysis was illustrated in Fig. 4.

#### Data extraction

Two investigators (Fa-liang Wang and Ming Yang) independently extracted data according to the terms and reached consensus for each trial. The following information was recorded: first author’s name, country of origin, sample size, MMTV-LV prevalence in breast carcinoma tissues. The information of all included studies was summarized in Table 5.

#### Meta-analysis

The meta-analysis consists of two parts. In the first part, we pooled the MMTV-LV infection rates in breast carcinoma reported in previous publications and this study. In the second part, we analyzed the correlation between MMTV-LV prevalence in human breast cancer and the distribution of different types of house mice in a global map (Supplementary Fig. 2) in a meta-regression model.

In the meta-analysis, Cochrane χ^2^ test was performed to examine the null hypothesis that the observed heterogeneity was random and the degree of heterogeneity was estimated using the statistic *I*^2^ = [(Q-*df)/*Q] × 100% (Q = Cochrane χ^2^, *df* = degrees of freedom). Due to the significant heterogeneity in this study, the DerSimonian and Laird method was adopted to pool the data in a random-effect model [61].

To determine the influence of the distribution of house mice on the prevalence of MMTV-LV in human breast cancer tissue, we performed a random-effects meta-regression. The proportion of heterogeneity attributable to distribution of house mice was estimated by comparing the between-studies component of variance in the null model (τ_0_^2^) with the estimate of τ^2^ for the model with the covariate of house mice distribution [(τ_0_^2^-τ^2^)/ τ_0_^2^]. *P* values of < 0.05 suggested that the factor involved in the meta-regression could partly explain the heterogeneity in the meta-analysis.

The publication bias was quantitatively assessed in Egger’s test [62]. When *P* value was less than 0.1, the publication bias was considered present. For other tests, *P* values of < 0.05 were considered statistically significant. The meta-analysis was carried out in STATA 12.0.

## Results

### Screening of MMTV-like env gene sequence in breast tumor DNA

The MMTV-like env gene sequence was screened in the genomic DNA from 119 breast cancer specimens, 50 breast fibroadenoma specimens and 4 human breast cancer cell lines. The house keeping gene GAPDH was amplified to confirm the integrity of all DNA samples. In the 119 breast cancer specimens, the PCR products of expected size were successfully amplified from 21 specimens. While in the 50 breast fibroadenoma tissues, only 2 samples were identified containing the sequence. Moreover, the sequence could also be detected in 2 cell lines, including Bcap-37 and MDA-MB-453 (Fig. 1).

### Sequence homology analysis of the PCR products

**Fig. 2:**
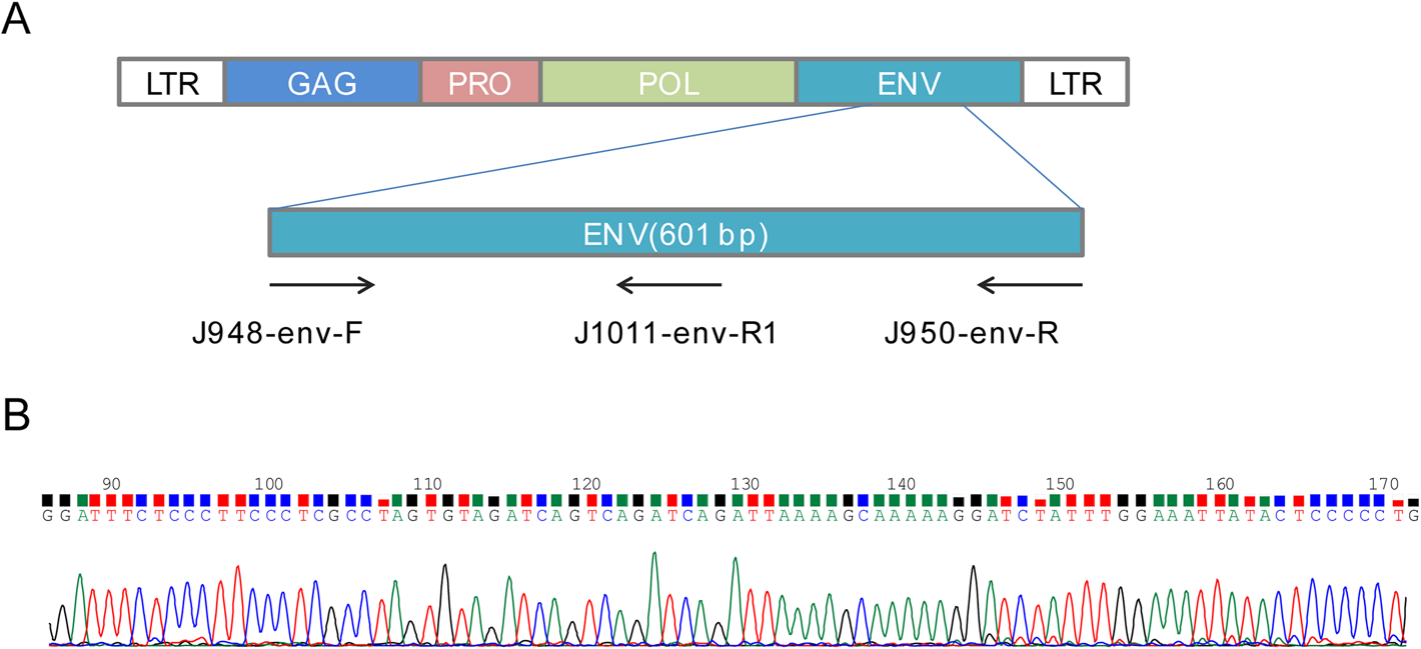
MMTV genome and the position of the primers. **A**. The outer primers used in the primary PCR were J948-env-F and J950-env-R. The inner primers used in the secondary PCR were J948-env-F and J1011-env-R1. All the primers were shown by arrows. **B**. DNA sequence of the MMTV-like env PCR product

**Fig. 3:**
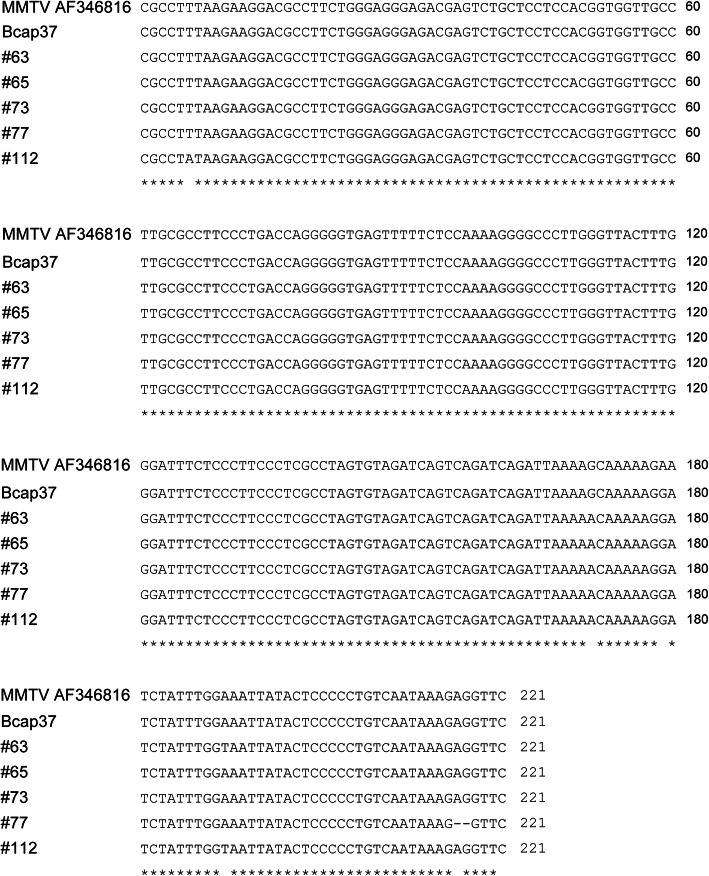
Multiple nucleotide alignment of the sequences of PCR products (marked by their respective numbers) with MMTV env sequences retrieved from GenBank database (No. AF346816). Sequences were aligned using DNAMAN, version 8. Stars show conserved sites along the alignment

To analyse the homology of PCR products to MMTV env gene, twelve PCR products randomly chosen from different specimens were sequenced. The sequence homology of the PCR products was 98–99% to the MMTV env sequence from the GeneBank (AF346816) (Figs. 2B and 3). So we reasoned that all of the specimens with PCR products were infected by MMTV-LV.

### Prevalence of MMTV-LV and its correlation with clinicopathological characteristics of patients

Consistent with our hypothesis, the positive rate of MMTV-LV env sequence was significantly correlated with sample sources (Southern China 5.71%, and Northern China 22.62%, *P* = 0.03).

The association between MMTV-LV prevalence and the clinical properties of the specimens was further analyzed with statistical approaches. We found that the prevalence of MMTV-LV env sequence in breast cancer tissues (17.65%) was much higher than that in breast fibroadenoma specimens (4.00%), with a *P*-value of 0.02. (Fig. 1D and Table 3). What’s more, the presence of MMTV-LV env sequence was significantly correlated with HER-2 expression in breast cancer tissues (Table 4). The proportion of samples with high HER-2 expression in MMTV-LV positive tissues (71.43%) was much higher than that in MMTV-LV negative tissues (43.88%) (Table 4, *P* = 0.02). No significant correlations were observed with the age of the patients, histological types, histological grades, lymph node status, tumor position, ER and PR expression in breast cancer tissues.

**Table 3:** Prevalence of MMTLV in breast cancer and breast fibroadenoma in Chinese women

Histopathological type	Number of MMTV-LV positive cases	Number of MMTV-LV negative cases	***P***-value^a^
Breast cancer (%)	21 (17.65)	98 (82.35)	**0.02**
Breast fibroadenoma (%)	2 (4.00)	48 (96.00)	

^a^*P* value was calculated by χ2 (two sided) and was considered to statistically significant for *P*<0.05. Bold values indicate significant correlation

**Table 4:** Characteristics of MMTLV positive and negative breast cancer in Chinese women

Variables	Number of MMTLV positive cases (%)	Number of MMTLV negative cases (%)	***P***-value ^a^
Ages (years)			0.68
<35	0 (0.00)	3 (3.06)	
35–50	10 (47.62)	49 (50.00)	
>50	11 (52.38)	46 (46.94)	
Sample source (province)			**0.03**
Northern China	19 (90.48)	65 (66.33)	
Southern China	2 (9.52)	33 (33.67)	
Histological type			0.61
Invasive ductal carcinoma	20 (95.24)	87 (88.78)	
Invasive lobular carcinoma	1 (4.76)	8 (8.16)	
Mucious Adenocarcinoma	0 (0.00)	3 (3.06)	
Histological grade ^b^			0.77
GradeI	8 (38.10)	40 (46.51)	
GradeII	11 (52.38)	38 (44.19)	
GradeIII	2 (9.52)	8 (9.30)	
Lymph node involvement			0.21
Positive	8 (38.10)	52 (53.06)	
Negative	13 (61.90)	46 (46.94)	
Position			0.82
Right	13 (61.90)	58 (59.18)	
Left	8 (38.10)	40 (40.82)	
Estrogen receptor			0.73
Positive	12 (57.14)	52 (53.06)	
Negative	9 (42.86)	46 (46.94)	
Progesterone receptor			0.54
Positive	12 (57.14)	63 (64.29)	
Negative	9 (42.86)	35 (35.71)	
HER-2			**0.02**
−/1+	6 (28.57)	55 (56.12)	
2+/3+	15 (71.43)	43 (43.88)	

^a^ *P* value was calculated by χ2 or Fisher’s tests and was considered to be statistically significant for *P*<0.05. Bold values indicate significant correlation

^b^ Histological grade was performed only in specimens with invasive ductal carcinoma

### Meta-analysis of MMTV-LV prevalence in breast cancer patients

**Fig. 4:**
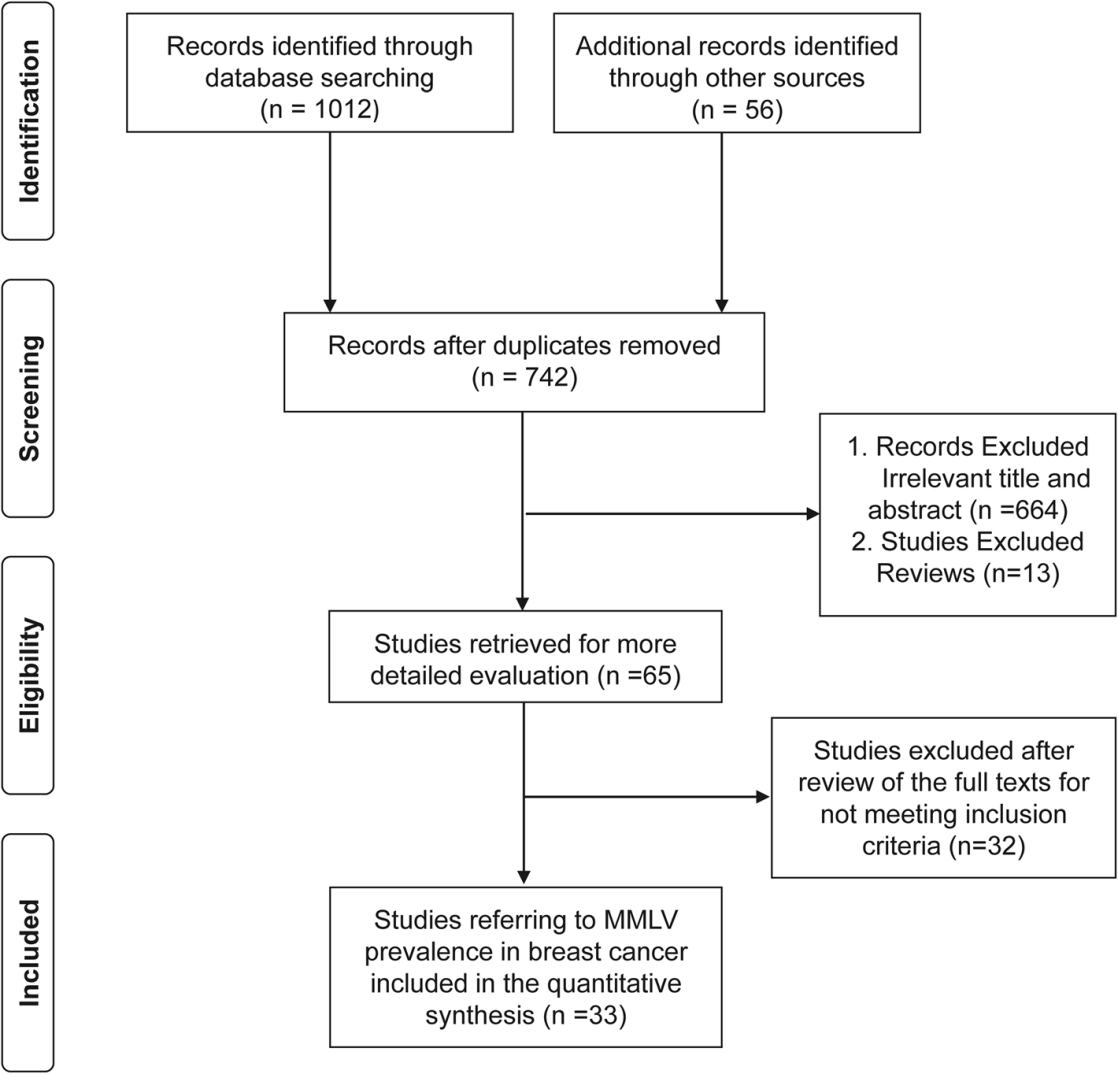
Flow diagram of the publication search strategy and assessment of studies identified for meta-analysis

As shown in Fig. 4, database searching identified 1012 records and a review of reference lists uncovered 56 additional records. After removing duplicate studies, 742 records remained, 664 of which were excluded after reviewing titles and abstracts. In the remaining 78 articles, 13 reviews were excluded. 65 records underwent full text review, and 33 studies met the inclusion criteria [20, 22, 46, 47, 55–57]. We also included the data in the above case-control study, which was divided into two data sets according to the sample sources (Hebei and Zhejiang), so there were 35 data sets in total (Table 5). A publication bias test was performed before the studies were pooled together. The result revealed no obvious publication bias (*P* = 0.499).

**Table 5:** Studies included for the meta-analysis

First Author	Year	Region	MMTV-LV Prevalence	Mouse species	Reference
Wang FL	2020	China (South)	2/35 (5.71%)	M.c (1)	–
Reza MA	2015	Iran	12/100 (12.00%)	M.c (1)	[55]
San TH	2017	Myanmar	1/58 (1.72%)	M.c (1)	[47]
Naushad W	2017	Pakistan	83/250 (33.20%)	M.c (1)	[22]
Ford CE	2004	Vietnam	1/161 (0.62%)	M.c (1)	[56]
Naushad W	2014	Pakistan	16/80 (20.00%)	M.c (1)	[20]
Shariatpanahi S	2017	Iran	19/59 (32.20%)	M.c (1)	[57]
Luo T	2006	China	22/131 (16.79%)	M.c/M.m (1.5)	[46]
Wang FL	2020	China (Nouth)	19/84 (22.62%)	M.m (2)	–
Nartey T	2017	Australia	9/25 (36.002%)	M.d (3)	[58]
Wang Y	2001	America	188/495 (37.98%)	M.d (3)	[31]
Nazar AMA	2014	Iraqi	22/38 (57.89%)	M.d (3)	[59]
Lawson JS	2006	Australia	21/59 (35.59%)	M.d (3)	[40]
Melana SM	2002	Argentine	23/74 (31.08%)	M.d (3)	[33]
Lawson JS	2018	Australia	12/45 (26.67%)	M.d (3)	[21]
Lawson JS	2004	Australia	20/42 (47.62%)	M.d (3)	[38]
Wang Y	2003	America	196/513 (38.21%)	M.d (3)	[35]
Ford CE	2003	Australia	19/45 (42.22%)	M.d (3)	[34]
Zapata-Benavides P	2007	Mexico	5/119 (4.20%)	M.d (3)	[42]
Lawson JS	2010	Australia	33/74 (44.59%)	M.d (3)	[44]
Mok MT	2008	Australia	28/50 (56.00%)	M.d (3)	[19]
Glenn WK	2012	Australia	39/50 (78.00%)	M.d (3)	[24]
Levine PH	2004	Tunisia	28/38 (73.68%)	M.d (3)	[39]
Melana SM	2001	Argentine	32/106 (30.19%)	M.d (3)	[16]
Etkind PR	2004	America	6/12 (50.00%)	M.d (3)	[36]
Faedo M	2004	Australia	50/128 (39.06%)	M.d (3)	[37]
Ford CE	2004	Australia	45/144 (31.25%)	M.d (3)	[17]
Mazzanti CM	2011	Italy	47/69 (68.12%)	M.d (3)	[25]
Pogo BG	1999	Italy	26/69 (37.68%)	M.d (3)	[29]
Wang Y	1995	America	181/465 (38.92%)	M.d (3)	[28]
Etkind P	2000	America	27/73 (36.99%)	M.d (3)	[30]
Al Dossary R	2018	Saudi Arabia	6/101 (5.94%)	M.d (3)	[23]
Naccarato AG	2019	Italy	17/56 (30.36%)	M.d (3)	[60]
Zammarchi F	2006	Italy	15/45 (33.33%)	M.d (3)	[41]
Hachana M	2008	Tunisia	17/122 (13.93%)	M.d (3)	[43]

Studies included for the meta-analysis

M.d: M. domesticus mouse; M.m: M. musculus mouse and M.c: M. castaneus mouse

According to the distribution of house mice [48, 49], we divided all the studies into three categories: M.c, M.m and M.d, which were numbered 1, 2 and 3, respectively. The characteristics of the included studies are summarized in Table 5. All the studies were region-based, in particular, 7 studies were based in M.c region (one data set from our case-control study) [20, 22, 47, 55–57], one study was based in M.c/M.m mixed region [46], one study was based in M.m region (our case-control study), and 26 studies were based in M.d region [16, 17, 19, 21, 23–25, 28–31, 33–44, 58–60].

**Fig. 5:**
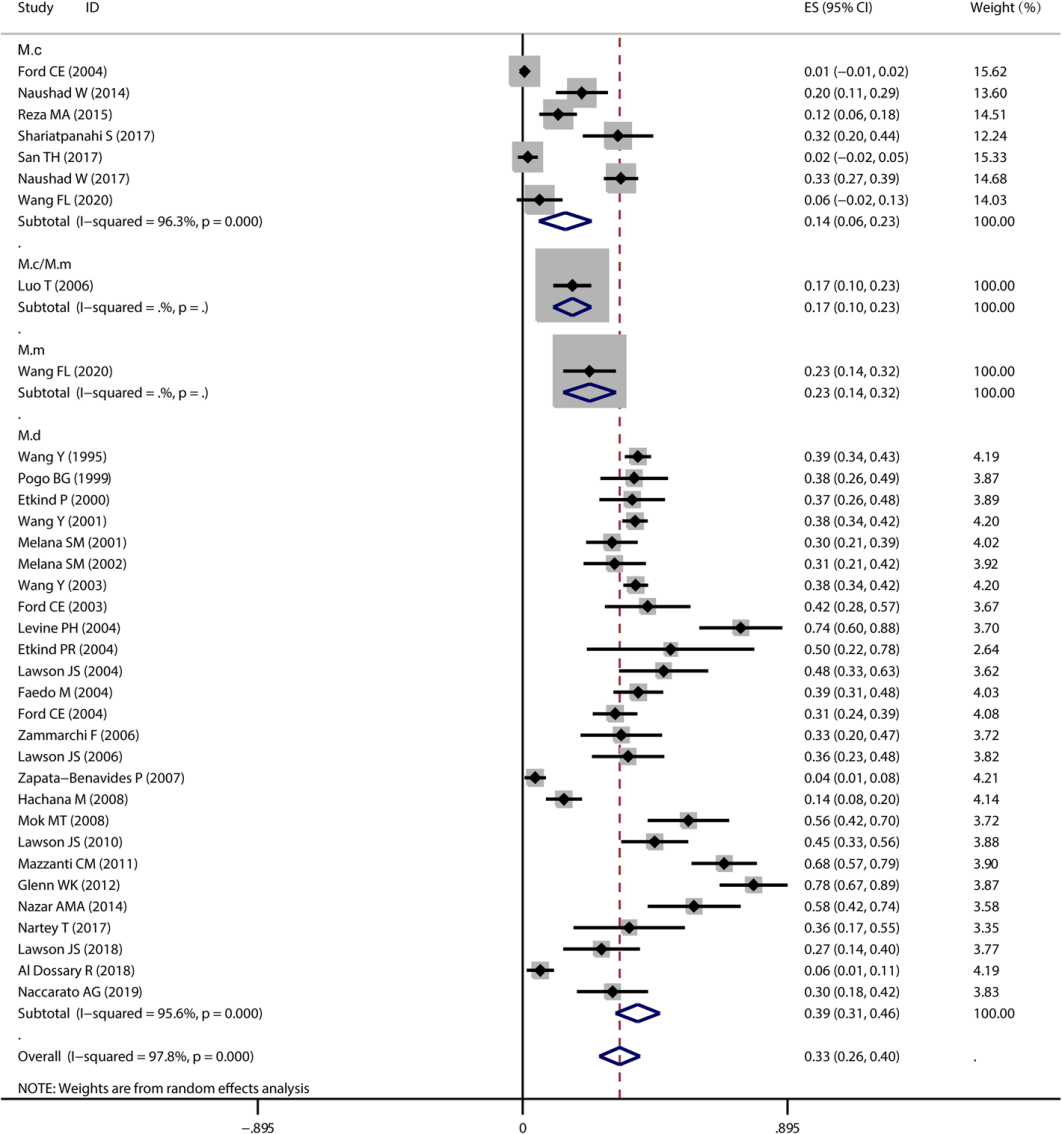
The meta-analysis of MMTV-LV prevalence in women breast cancer tissue. The data sets were divided in four subgroups (M.c, M.c/M.m, M.m and M.d) and analysed in a random effects model

MMTV-LV prevalence ranged from 1 to 78% in breast carcinoma tissue and the overall positive rate was 33% (95%CI: 26–40%). There was a statistically significant heterogeneity between the included studies (*I*^*2*^ = 97.8%, Cochran’s Q test: *P* < 0.05). In the subgroup analysis, it was found that MMTV-LV prevalence was lowest in M.c region (14, 95%CI: 6–23%) and highest in M.d region (39, 95%CI, 31–46%). There were statistically significant differences between the subgroups (*P* < 0.05) (Fig. 5).

**Fig. 6:**
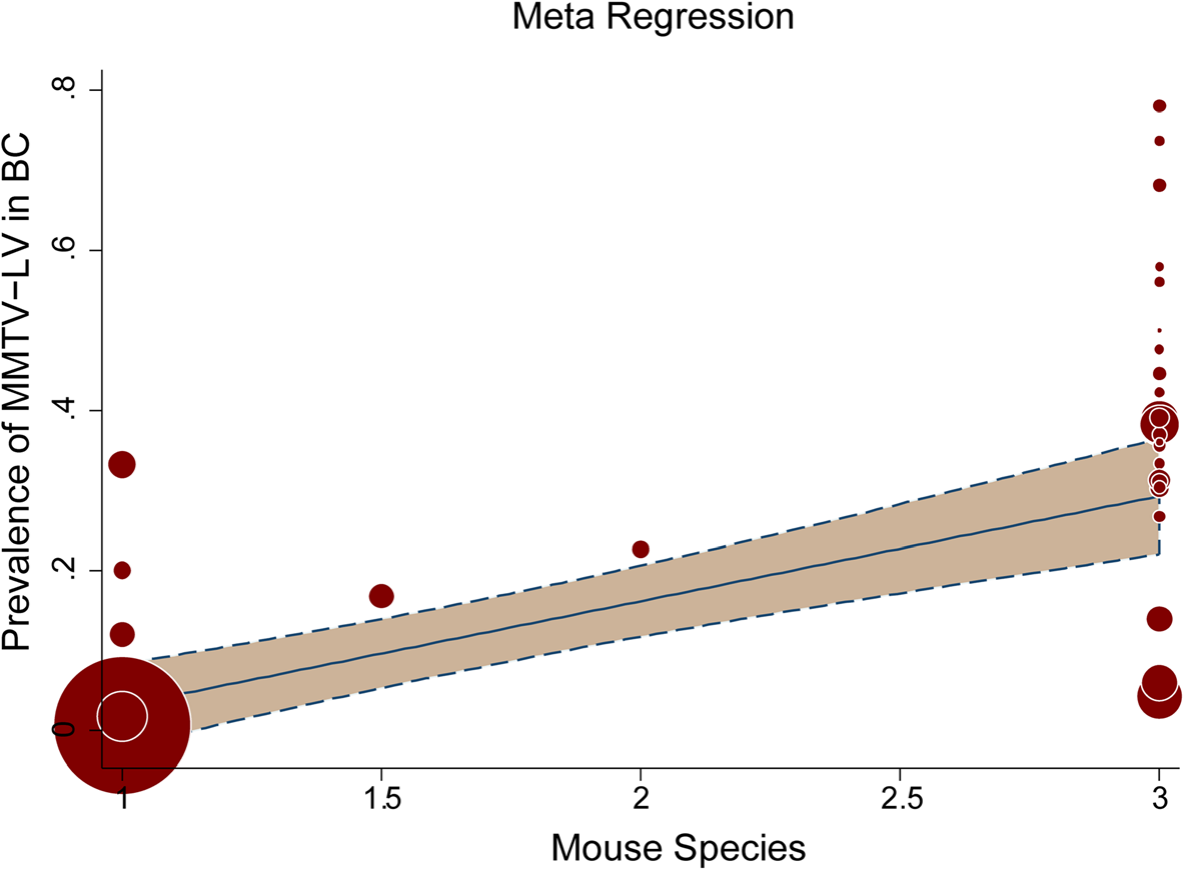
Meta-regression analysis. In this model, the dependent variable is prevalence of MMTV-LV, while the covariant is the mouse species. X-axis represents different house mice species (1: M.c, 1.5: M.c/M.m, 2: M.m, 3: M.d). Y-axis denotes the prevalence of MMTV-LT in breast cancer in Chinese women

To confirm the effect of house mice distribution on MMTV-LV prevalence, a meta-regression analysis was performed. The analysis result demonstrated that 27.31% variance between studies coming from different countries could be explained by the distribution of house mice, which was a crucial factor that affected the prevalence of MMTV-LV significantly (*P* < 0.05) (Fig. 6).

## Discussion

Since the role and mechanism of viruses on cancer development were proposed in the 1970s and 1980s, there has been an avalanche of studies on the correlation between MMTV-LV and human breast carcinogenicity. Closely following the findings, studies discovered MMTV-like antigens or viral particles in human breast or breast cancer cells. Beyond that, immune responses to MMTV-LV antigens had also been detected in women with breast cancer [63–65]. However, some MMTV-LV sequence screening studies unexpectedly revealed that the detected DNA was highly homologous to human endogenous retrovirus (HERV), thereby hindering the development of related studies [66]. Until recent decades, the widespread application of PCR technology allows scientists to use primers from selected regions of genes with low homology to known HERV to detect MMTV-like DNA sequences in human breast cancer tissues [28]. Therefore, renewed interest in the viral etiology of breast cancer has been aroused.

In the first part of our study, we screened MMTV-LV env sequence in Chinese women breast cancer tissues, breast fibroadenoma specimens and human breast cancer cell lines. Except for several random mutations, the sequences of the PCR products showed 98–99% homology with the MMTV env sequence (Fig. 3). This signifies that house mouse, an important carrier of MMTV, may be a zoonotic source of MMTV-LV in human breast tumor. As a supportive argument for early human exposure, Stewart et al. [49] indicated that mouse fecal particles contaminated the stored grains, so the MMTV might enter the human digestive tract and then infected humans. In addition, cats might be infected with MMTV carried by mice and then spread the virus to humans [67]. Moreover, MMTV can also transmit through saliva inter-human [68]. MMTV-LV was ever found in the breast cancer tissues of father, mother and daughter, suggesting the possibility of MMTV-LV as an etiological agent which is related to familial breast cancer [69].

In the case-control study, the results indicated that the prevalence of MMTV-LV in breast cancer specimens (17.65%) was much higher than that in breast fibroadenoma tissues (4%) (Fig. 1D and Table 3), implying that infection of MMTV-LV may play an important role in breast carcinogenesis.

The long terminal repeat (LTR) sequence of MMTV was found to contain all the enhancer and promoter elements. Proviral DNA can integrate into the vicinity of cellular proto-oncogenes and activate their transcription, which is postulated to be the carcinogenic mechanism of MMTV [70]. Due to the non-specific integration site of MMTV in genes [71], the higher concentration of the virus, it is more likely that the proviral DNA will integrate into the vicinity of the proto-oncogene. In fact, several mammalian oncogenes such as Wnt gene family were discovered just because of their correlation with MMTV [9]. Therefore, the incidence and latency of breast cancer are proportional to the burden of MMTV [72]. Besides, the env protein also involves in transformation process of mammary epithelial cells mediated by MMTV, because MMTV-induced breast tumor can be reduced by the mutation of the env protein, even the virus load is at high levels [73]. MMTV-LV, containing LTR and env sequences, shares 95% homology with MMTV and has the potential to express, replicate and integrate into the genome of host cells [74]. MMTV-LV may trigger the occurrence of breast cancer with similar mechanism.

In the analysis of the correlation between prevalence of MMTV-LV and clinicopathological characteristics, we found that the presence of MMTV-like env sequences in breast carcinoma specimens was associated significantly with sample source and HER-2 expression (Table 4).

HER2 is a known proto-oncogene and it is overexpressed in about 30–50% of human breast cancer [75, 76]. The overexpression of HER-2, predicting a poor prognosis, plays a key role in breast cancer development and metastasis. Thus, HER-2 has become an important target in breast cancer treatment [77, 78]. In vitro experiments have showed that Epstein-Barr virus infection was related with increased expression and activation of HER2 signaling cascades, which could be blocked by trastuzumab [79]. Our finding suggests that the role of MMTV-LV in human breast cancer may be also correlated to the HER2 oncogenic pathway, but it remains to be confirmed in further molecular mechanism research.

With regard to sample sources, we discovered the prevalence of MMTV-LV in Chinese breast cancer is correlated with the distribution of house mice. According to the studies reported previously [48, 49], Zhejiang and Hebei, which respectively locate in Southern and Northern China, were distributed with different kind of house mice (M.c and M.m, respectively). The different prevalence of MMTV-LV in Southern and Northern China may due to the different viral burden of MMTV in mice [49].

In order to confirm the association between the prevalence of MMTV-LV in breast cancer tissues and the distribution of house mice worldwide, we then performed a meta-analysis in subgroups and a meta-regression analysis. We found that the MMTV-LV prevalence in breast cancer was increasing from M.c region, M.m region to M.d region. The distribution of house mice could explain 27.31% of the difference in the MMTV-LV prevalence in breast cancer specimens from different regions.

To sum up, the distribution of house mice in the world may partly account for the geographical variation in breast cancer incidence worldwide. Study of Hunter et al. showed that high-fat diet and xeno-oestrogens intake were considered risk factors for breast cancer [80]. However, the diet of circumpolar Inuit is rich in saturated fat and contaminated with high doses of xeno-oestrogens [81], but the incidence of breast cancer is low [82]. More interestingly, it is generally agreed that environmental changes can lead to an increased risk of breast cancer [83]. When people migrate to areas with higher breast cancer incidence, their risk of breast cancer gradually increases. For instance, the migration of Japanese to the United States [84], and South Asians to Britain [85] demonstrated that the incidence of breast cancer had increased over decades. Among these areas, a remarkable difference is the distribution of M.c, M.m and M.d with different MMTV burden, so it may be the crucial environmental factor that explains the geographic variation in human breast cancer incidence.

## Conclusion

The results of this study suggest that MMTV-LV infection may increase the risk of breast cancer. Besides, MMTV-LV prevalence is significantly associated with sample source and HER2 expression, but there is no significant correlation between MMTV-LV prevalence and age of patients, histological grade, histological type, lymph node involvement, tumor position or expression of ER and PR in breast cancer tissues. In the meta-analysis, we find that MMTV-LV prevalence in breast cancer is dependent on the distribution of M.c, M.m and M.d. The results of this study support the zoonotic hypothesis that MMTV-LV is transferred from domestic mice to human and is a crucial risk factor for human breast cancer. These findings may provide a potential avenue in breast cancer prevention, diagnosis and treatment.

## Supplementary Information

**Additional file 1.**

## Data Availability

All data supporting the findings of this study are available with the corresponding authors.

## Abbreviations

MMTV-LV: Mouse Mammary Tumor Virus-Like Virus
ER: Estrogen receptor
PR: Progesterone receptor
M. d: M. domesticus mouse
M.m: *M. musculus* mouse
M.c: M.castaneus mouse
