# Supplementary material for: Prevalence and characteristics of mouse mammary tumor virus-like virus associated breast cancer in China
Source: Infect Agent Cancer. 2021 Jun 26;16:47. doi: 10.1186/s13027-021-00383-2 (PMC8235620; doi:10.1186/s13027-021-00383-2)
Supplement: Supplementary file 1 — Additional file 1. [file 13027_2021_383_MOESM1_ESM.docx]

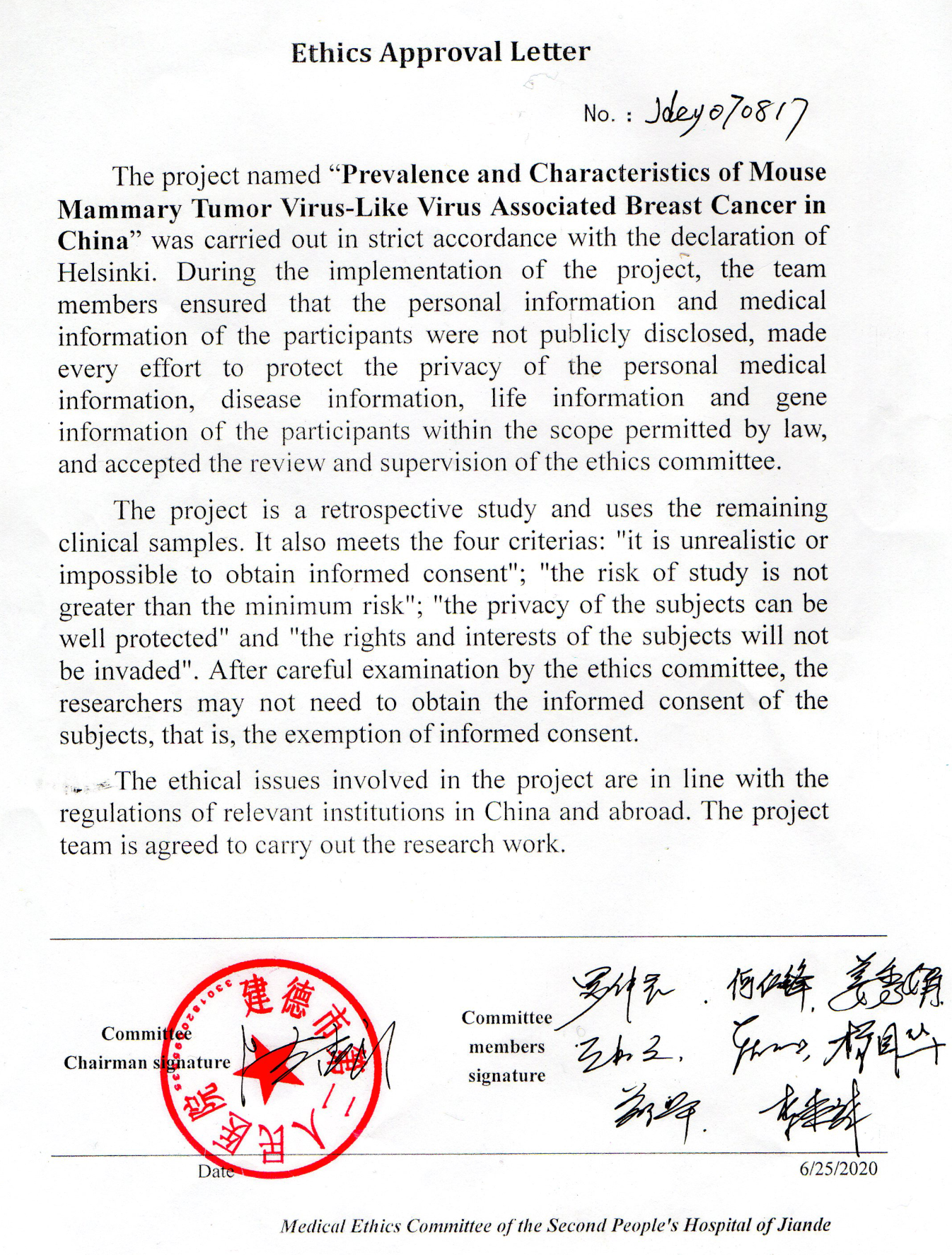


Figure1. The ethics approval letter of research ethics committee


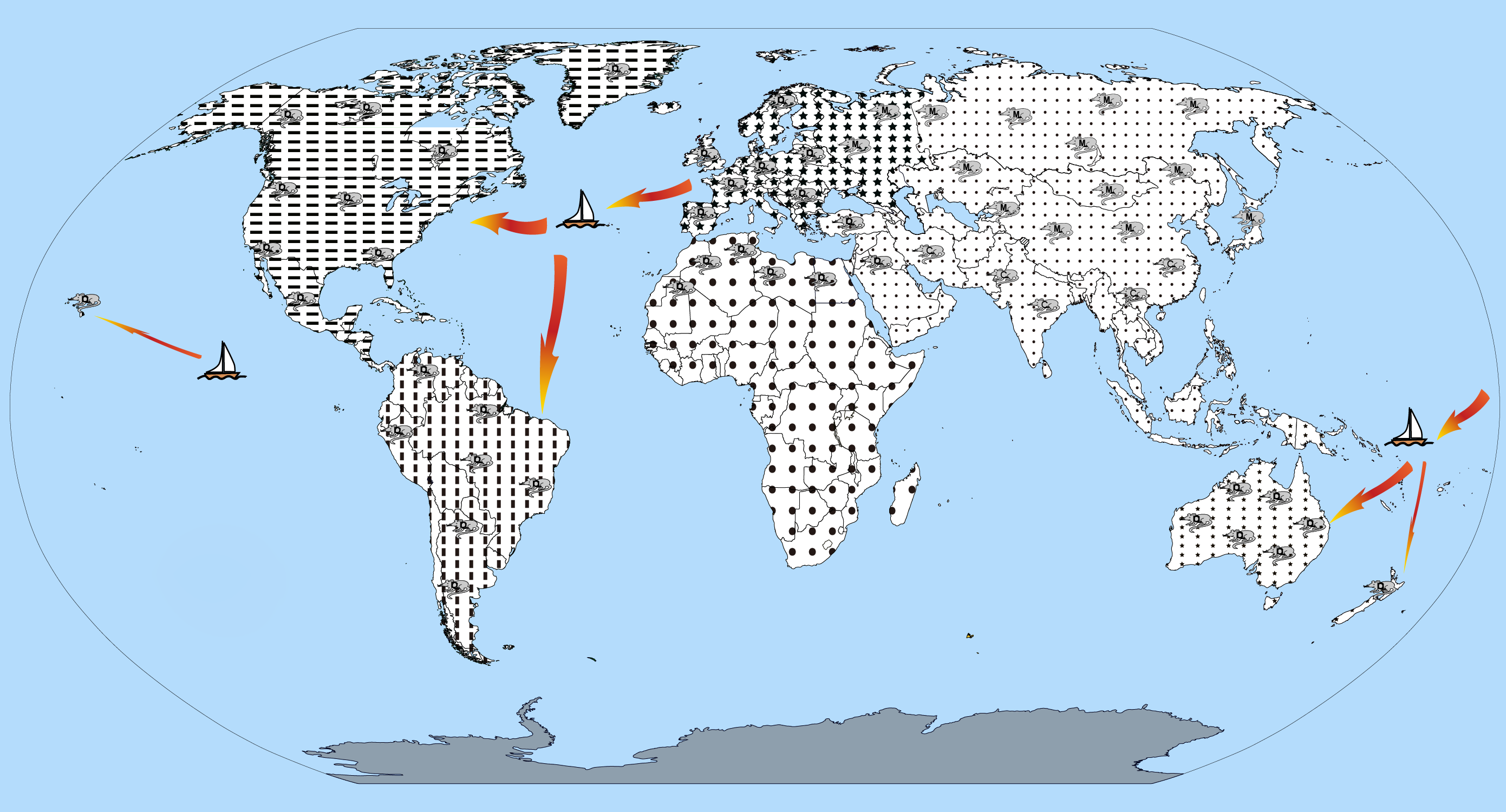


Figure2. The distribution of M.d, M.c and M.m in the world ( D: M.d, C: M.c, M: M.m)
